# Mitochondrial DNA sequence of the horseshoe crab *Tachypleus gigas*

**DOI:** 10.1080/23802359.2021.1930213

**Published:** 2021-05-23

**Authors:** Maria E. Sarmiento, Kai Ling Chin, Nyok Sean Lau, Ismail Aziah, Mohd Nor Norazmi, Armando Acosta, Noraznawati Ismail, Nik Soriani Yaacob

**Affiliations:** a School of Health Sciences, Universiti Sains Malaysia, Health Campus, Kelantan, Malaysia; b Faculty of Medicine and Health Sciences, Universiti Malaysia Sabah, Kota Kinabalu, Malaysia; c Centre for Chemical Biology, Universiti Sains Malaysia, Pulau Pinang, Malaysia; d Institute for Research in Molecular Medicine, Universiti Sains Malaysia, Health Campus, Kelantan, Malaysia; e Institute of Marine Biotechnology, Universiti Malaysia Terengganu, Terengganu, Malaysia; f Department of Chemical Pathology, School of Medical Sciences, Universiti Sains Malaysia, Health Campus, Kelantan, Malaysia

**Keywords:** *Tachypleus gigas*, mitochondrial genome, phylogenetic analysis, whole genome sequencing, Terengganu, Malaysia

## Abstract

This paper reports on the complete mitochondrial (mt) genome of a horseshoe crab, *Tachypleus gigas* (*T. gigas*), in Kuala Kemaman, Terengganu, Malaysia. Whole-genome sequencing of hemocyte DNA was performed with Illumina HiSeq system and the generated reads were *de novo* assembled with ABySS 2.1.5 and reassembled using mitoZ against *Carcinoscorpius rotundicauda* and *Limulus polyphemus*, resulting in a contig of 15 Kb. Phylogenetic analysis of the assembled mt genome suggests that the *Tachypleus gigas* is closely related to *Tachypleus tridentatus* than to *Carcinoscorpius rotundicauda.*

## Introduction

Horseshoe crabs (HSCs) are important species in selected marine ecosystems, being the source of protein for other marine species and contributing as bioturbators to the rearrangement and aeration of marine sediments. HSCs also have an important role in the biomedical and pharmaceutical industries, and an important subject of research from the biological, evolutionary, and ecological standpoints (Manca et al. 2017; Krisfalusi-Gannon et al. 2018). A combination of factors such as environmental changes and uncontrolled economical exploitation threaten the survival of HSCs which encourages the genetic research on these species to better understand their mechanisms of adaptation, genetic diversity, and immune defensive system.

HSCs are marine, brackish water arthropods of the phylum Arthropoda, subphylum Chelicerata, order Xiphosura, suborder Xiphosurida, family Limulidae, and subfamily Tachypleinae. Based on their unique and limited morphological evolution, HSCs are considered as ‘living fossils.’ The evolutionary history of HSCs dates around 450 million years ago (Zhou et al. 2020). HSCs present some ordinary traits of crustaceans but are more associated with arachnids such as spiders and scorpions (Chen et al. 2016). At present, there are only four known HSC species (Gong et al. 2019). The *Carcinoscorpius rotundicauda* (*C. rotundicauda)* (mangrove) species is distributed across South and Southeast Asia, mainly in Thailand (Kanchanapongkul 2008), Malaysia, Eastern India, Bangladesh, Cambodia, southern and northern Vietnam, southern China, Sumatra, Java, and Borneo (Vestbo et al. 2018); *Limulus polyphemus* (*L. polyphemus*) (Atlantic or American) lives in the Gulf of Mexico and in the American Atlantic coast (Zhu et al. 2020); *Tachypleus tridentatus* (*T*. *tridentatus)* (Chinese, Japanese or tri-spine) is distributed in Southeast and East Asia mainly in Sabah, Malaysia (Mohamad et al. 2016), southern Japan, China, Taiwan, northern Vietnam, Philippines, Island of Borneo and Java (Vestbo et al. 2018) and *Tachypleus gigas* (*T. gigas*) (Indo-Pacific, Indonesian, Indian or southern), which is found in South and Southeast Asia in places such as Malaysia, in the States of Pahang (Razak et al. 2017) and Terengganu (Rozihan and Ismail 2012), as well as in Thailand and India (Vestbo et al. 2018).

Genomes and transcriptomes from different HSC species (*L. polyphemus*, *T*. *tridentatus*, and *C. rotundicauda*) have been described previously (Ding et al. 2005; Battelle et al. 2016; Chen et al. 2016; Chesmore et al. 2016; Gong et al. 2019; Liao et al. 2019; Lou et al. 2020; Shingate et al. 2020; Zhou et al. 2020). However, information about the genome of *T. gigas* has been scarce, which has limited the knowledge on the biology of this species. In this regard, a recent study reported the genome sequence of a *T. gigas* specimen from Singapore (Shingate et al. 2020). Our current study, therefore, complements this recent report by providing the mitochondrial (mt) genome data of a specimen of the same species from a different geographical habitat.

The study of the mt DNA sequence of different HSCs species has been a focus of scientific interest due to its importance to establish their evolutionary and phylogenetic relationship (Baek et al. 2014). The mt DNA sequence of *L. polyphemus, C. rotundicauda,* and *T. tridentatus* have been reported previously (Lavrov et al. 2000; Baek et al. 2014). In this study, we reported the mt DNA sequence of a *T. gigas* specimen from Terengganu, Malaysia, whose genome has been sequenced by our group, together with a phylogenetic study based on the mt protein-coding genes which will contribute to the better understanding of genomic features of this endangered species.

## Materials and methods

### Sample collection

An adult HSC was purchased from the fishermen who collected it from the coastal area in Kuala Kemaman, Terengganu, Malaysia. The HSC was transported to the Universiti Malaysia Terengganu (UMT) Hatchery at night to avoid direct heat from the environment. HSCs are sensitive to heat and this would induce stress. The HSC was allowed to acclimatize for 3 days and was cleaned thoroughly before sample collection. The hemolymph was collected using pyrogen-free apparatus in sterile condition in a Biological Safety Cabinet Class II (ESCO, USA) following patented techniques (MY-155541-A) from Makmal Belangkas, UMT. Hemolymph (2 mL) was drawn out and the HSC was transported back to the sea after sample extraction.

### DNA purification

DNA from the hemocytes was extracted using a DNA purification kit (QIAamp DNA Blood Mini Kit, Qiagen) according to the manufacturer’s instructions. Briefly, 1 mL of fresh hemolymph was added to 40 ml of 3% NaCl contained in 50 mL sterile-polypropylene-centrifuge tubes and mixed thoroughly. The tube was centrifuged at 2000 × *g* for 5 min, then the supernatant was discarded. The genomic DNA was extracted from the cell pellet. DNA integrity and quantification were determined by agarose gel electrophoresis, Nanodrop and Qubit™ (Invitrogen).

### Library construction, quality control, and sequencing

A total amount of 500 ng DNA was used as input material for the sample preparation. Sequencing libraries were generated using NEBNext^®^ Ultra II DNA Library Prep Kit (New England Biolabs, England) according to the manufacturer's recommendations and index sequences were added to the genomic libraries. The genomic DNA was randomly fragmented to a size of 350 bp by Covaris cracker (Covaris, USA), before the DNA fragments were end-polished, A-tailed, and ligated with the full-length adapter for Illumina sequencing with further PCR amplification. The PCR products were purified (AMPure XP system) and the libraries were analyzed for size distribution by Agilent 2100 Bioanalyzer and quantified using real-time PCR. The qualified libraries were loaded into Illumina HiSeq sequencer after pooling according to its effective concentration and expected data volume.

### Quality control and mitochondrial assembly

Paired-end Illumina sequences were first removed of sequence adaptors and reads with low-quality scores using bbduk of the BBTools Packages (https://jgi.doe.gov/data-and-tools/bbtools/). Reads were assembled *de novo* using ABySS 2.1.5 (Jackman et al. 2017). To determine the presence of mt genome in the Abyss kmer72 assembly, the mt genome of *C. rotundicauda* (NC_019623) was used to search against the kmer72 assembly using blastn. The search recovered a contig of approximately 14 Kb, but this contig is linear and less than the size of the reference mt genome of approximately 15 Kb. The Abyss contigs were used as input for reassembly with mitoZ (Meng et al. 2019) by comparing to *C. rotundicauda* and *L. polyphemus* (NC_003057.1). The open reading frames were predicted with reference to the invertebrate mt translational table, and annotation was done using MITOS1 software (http://mitos.bioinf.uni-leipzig.de/index.py) (Bernt et al. 2013). A pairwise comparison of mitogenome sequences between *T. gigas* and *C. rotundicauda* was performed using BLASTN 2.9.0.

### Phylogenetic analysis

Mt protein-coding genes nucleotide sequences from different organisms: *Ixodes hexagonus* (AF081828), *T. tridentatus* (JQ739210 and FJ860267), *T. gigas* (MT241669), *C. rotundicauda* (JX437074 and JQ178358) and *L. polyphemus* (NC_003057 and JX983598), were selected to determine the phylogenetic analysis of *T. gigas.*

The nucleotide sequence alignment was performed using ClustalW software and a maximum likelihood tree was constructed using MEGA X with 1000 bootstrapping (Kumar et al. 2018).

## Results and discussion

### Mitochondria genome

The sequencing reads first *de novo* assembled using Abyss and the Abyss contigs were then used as input for the mitoZ (Meng et al. 2019) reassembly against *C. rotundicauda* and *L. polyphemus*. The final assembled contig result showed a genome size of 15 Kb and exhibited similar gene organization compared to *C. rotundicauda* and *L. polyphemus*. Gene annotation with MITOS1 showed the mitochondrial genome contained 13 protein-coding genes (Supplementary Figure 1).

### Phylogenetic analysis

Several phylogenetic studies have been focused on HSCs, however, the relationship between *L. polyphemus* and the Asian HSC species is not well defined. A fragment of mt cox1 (or COI) gene, which is a ‘DNA barcode’ used as a universal marker for species identification (Pentinsaari et al. 2016), either alone or in combination with other markers had been used for HSC phylogenetic studies (Avise et al. 1994; Kamaruzzaman et al. 2011; Obst et al. 2012; Baek et al. 2014; Dhar et al. 2016; John et al. 2016). HSC phylogenetic studies using other markers have also been reported (Sekiguchi and Sugita 1980; Shishikura et al. 1982; Miyata et al. 1984; Iwanaga and Kawabata 1998; Ismail and Sarijan 2011; Lamsdell 2016).

**Figure 1.**
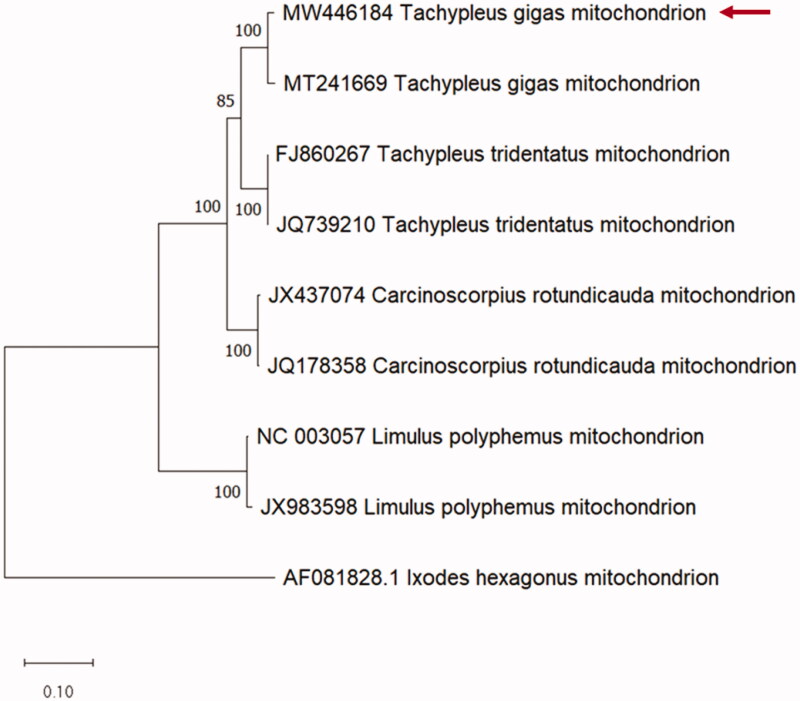
Comparative phylogenetic tree of *Tachypleus gigas* (Class: Merostomata; Family: Limulidae). Maximum likelihood tree of mitochondrial-protein coding genes using MEGA X with 1000 bootstrapping. *Ixodes hexagonus* (AF081828) was used as an out group. Red arrow: Mitochondrial DNA sequence (MW446184) obtained in this study.

In the present study, the phylogenetic position of *T. gigas* among the HSC species was analyzed using whole mt protein-coding genes with *L. hexagonus* as the outgroup. *T. gigas* in this study was clustered together with the other *T. gigas* sequence (MT241669) and showed a closer relationship with *T. tridentatus* than with *C. rotundicauda* (Figure 1). Our findings are consistent with previous phylogenetic reports (Obst et al. 2012; Dhar et al. 2016; John et al. 2016; Lamsdell 2016). A recent study using a genome-wide protein dataset supports the *Tachypleus* monophyly (Shingate et al. 2020).

However, in contrast to our results, Avise et al. (1994) and Kamaruzzaman et al. (2011) showed a closer relation of *T. gigas* with *C. rotundicauda* than with *T. tridentatus*.

Iwanaga and Kawabata (1998) and Shishikura et al. (1982) conducted phylogenetic studies based on coagulogen and peptide C, respectively, and a closer relationship between *T. tridentatus* and C. *rotundicauda* compared to *T. gigas* was reported.

Baek et al. (2014) reported a phylogenetic study based on whole mt genomes, which did not include *T. gigas* in their analysis.

Our study found, after pairwise mitogenome similarity comparison, mt DNA sequence differences (97% of identity) compared to the mt DNA sequence reported by Shingate et al. (2020), which could be related to the influence of the different geographic ecosystems. The specimen studied by Shingate et al was obtained from East Coast Park, Singapore, while our specimen was collected from Kuala Kemaman, Terengganu, Malaysia.

Genetic differences have been found in the same HSC species, including *T. gigas*, belonging to different geographical locations, which could be explained by multiple factors such as the limited migratory capacity of HSCs that influence the gene flow between populations, among others (Xia 2000; Ismail and Sarijan 2011; Rozihan and Ismail 2011; Obst et al. 2012; Adibah et al. 2015; Liew et al. 2015; Periasamy et al. 2017).

In summary, this study presents the whole mt DNA sequence of a *T. gigas* specimen from Terengganu, Malaysia, and suggests a close phylogenetic relationship between *T. gigas* and *T. tridentatus*, compared to *C. rotundicauda.*

## Data Availability

The data that support the findings of this study are openly available in NCBI Genbank database at (https://www.ncbi.nlm.nih.gov) with the accession number (MW446184).
